# CaMKK2 in Central Nervous System Disorders: Context-Dependent Signaling and Therapeutic Challenges

**DOI:** 10.3390/cells15212012

**Published:** 2026-11-05

**Authors:** Jia-Xi Zhang, Yi-Kai Zou, Xian-Guang Lin, Shao-Hua Qi, Jia-Wei Min

**Affiliations:** 1 College of Biomedical Engineering, South-Central Minzu University, Wuhan 430074, China; 15927777485@163.com (J.-X.Z.); 13204999331@163.com (Y.-K.Z.); 2012115@mail.scuec.edu.cn (X.-G.L.); 2 Ting Tsung and Wei Fong Chao Center for BRAIN, Houston Methodist Academic Institute and Weill Cornell Medicine, Houston, TX 77030, USA; qsh7950@gmail.com; 3 Hubei Key Laboratory of Medical Information Analysis & Tumor Diagnosis and Treatment, Wuhan 430074, China; 4 Key Laboratory of Cognitive Science, State Ethnic Affairs Commission, Wuhan 430074, China

**Keywords:** CaMKK2, calcium signaling, central nervous system disorders, context-dependent signaling, therapeutic targeting, AMPK, neurodegenerative disease

## Abstract

**Highlights:**

**What are the main findings?**

- CaMKK2 links Ca^2+^/CaM signaling to CaMK1, CaMK4, and AMPK pathways, with additional context-dependent associations involving Akt signaling.
- The effects of CaMKK2 signaling depend on cell type, downstream pathways, disease context, and, in some cases, the timing of intervention.

**What are the implications of the main findings?**

- Strategies targeting CaMKK2 should account for differences between central nervous system (CNS) disorders and cell types, rather than relying on uniform inhibition.
- The limited selectivity of current kinase inhibitors, their uncertain CNS exposure, and insufficient target-engagement evidence underscore the need for better pharmacological tools and cell-specific validation.

**Abstract:**

Ca^2+^/calmodulin-dependent protein kinase kinase 2 (CaMKK2) couples calcium signaling to metabolic, inflammatory, and transcriptional pathways and has been implicated in a broad range of central nervous system (CNS) disorders. However, its effects vary across disease settings, and the mechanisms underlying this context dependence remain poorly defined. This narrative review examines genetic, transcriptomic, biochemical, cellular, and in vivo evidence on the roles of CaMKK2 across CNS disorders, with emphasis on signaling pathways, cell type-specific functions, and implications for therapeutic targeting. CaMKK2-associated pathways influence neurovascular responses, synaptic function, protein homeostasis, inflammation, and tumor progression through signaling networks involving AMPK, CaMK1, CaMK4, and Akt. CaMKK2 can exert distinct, and in some settings opposing, effects depending on cell type, downstream pathway engagement, intervention timing, and pathological context. Pharmacological studies remain limited by incomplete selectivity, insufficient characterization of CNS pharmacokinetics, and context-dependent biological effects. Moreover, much of the mechanistic evidence derives from experimental models, and validated biomarkers for clinical translation remain limited. Overall, CaMKK2 emerges as a context-dependent signaling node linking calcium signaling to metabolic and inflammatory pathways in CNS disorders, with therapeutic potential but substantial challenges for therapeutic translation.

## 1. Introduction

Ca^2+^/calmodulin-dependent protein kinase kinase 2 (CaMKK2), also known as CaMKKβ in earlier studies, is a serine/threonine kinase that couples intracellular Ca^2+^/calmodulin signals to downstream metabolic and transcriptional programs. Initially characterized as an intracellular signaling component, CaMKK2 is now recognized as a broader regulator of CNS function [1,2]. Studies have implicated CaMKK2 in long-term memory formation, mood- and anxiety-related behaviors, and energy metabolism [3,4,5,6]. However, these observations remain fragmented and, in some settings, appear contradictory. CaMKK2-associated signaling has been linked to both protective and deleterious effects, depending on cell type, downstream pathway engagement, intervention timing, and pathological context. Such context dependence, together with the diversity of downstream signaling networks regulated by CaMKK2, has made it difficult to define a unified role for CaMKK2 in CNS pathology. Growing interest in signaling pathways that integrate metabolic, inflammatory, and synaptic processes has further highlighted CaMKK2 as a potential regulatory node [7]. Nevertheless, its translational relevance remains uncertain. Much of the evidence comes from preclinical models, and important questions concerning cell type-specific functions, context-dependent pathway engagement, and the feasibility of therapeutic modulation remain unresolved.

This review examines the roles of CaMKK2 across CNS disorders, focusing on how its functions vary with cell type, pathological context, downstream signaling, and, where evidence is available, intervention timing. It also considers differences in CaMKK2-associated signaling among neurons, glial cells, neurovascular cells, and tumor-associated cellular compartments, while distinguishing direct evidence of CaMKK2-dependent mechanisms from indirect evidence based on downstream pathways. The review also discusses the contexts in which CaMKK2 modulation may have therapeutic value.

Previous reviews have covered the molecular regulation of CaMKK2 and its roles in metabolism, cancer, and neural function [1,8]. This review instead focuses on how CaMKK2-associated signaling differs across CNS disorders and examines the evidence for its reported protective and detrimental effects. We distinguish findings based on direct CaMKK2 manipulation from those involving downstream signaling pathways, while considering the influence of cell type, intervention timing, and pharmacological selectivity. Comparisons across disease contexts are used to highlight unresolved mechanistic questions and identify the experimental evidence needed to assess CaMKK2 as a potential therapeutic target.

## 2. Literature Search and Review Strategy

This narrative review was based on literature identified through searches of PubMed and Web of Science, with searches conducted up to October 2026. The search strategy combined terms related to CaMKK2, including “CaMKK2,” “CaMKKβ,” and “calcium/calmodulin-dependent protein kinase kinase 2,” with terms describing relevant CNS disorders and pathological processes, including ischemic stroke, neonatal hypoxic–ischemic brain injury, psychiatric disorders, neurodegenerative diseases, glioblastoma, inflammation, oxidative stress, and apoptosis. Additional searches included terms related to pharmacological intervention, such as “inhibitor,” “pharmacology,” “therapeutic targeting,” and “STO-609,” to identify studies addressing the therapeutic potential and limitations of CaMKK2 modulation.

Original research articles reporting genetic, biochemical, cellular, animal, or human evidence were prioritized. Review articles were used mainly to provide background information and identify relevant studies. Particular attention was given to studies using genetic manipulation of CaMKK2, complementary pharmacological approaches, or functional validation of downstream mechanisms. Findings involving AMPK or other associated pathways without direct CaMKK2 manipulation were treated as supporting evidence or as a basis for further investigation, rather than proof of CaMKK2 dependence. When results differed between studies, we compared the experimental models, cell types, intervention timing, and outcome measures before interpreting the differences. Conflicting findings were retained, and discrepancies were noted when the available evidence did not provide a clear explanation. Transcriptomic, bioinformatic, and clinical association studies were interpreted with consideration of their methodological limitations. The aim was to provide a critical narrative synthesis rather than a systematic review or meta-analysis.

## 3. Molecular Architecture and Regulatory Mechanisms of CaMKK2

### 3.1. Molecular Structure of CaMKK2

**Figure 1:**
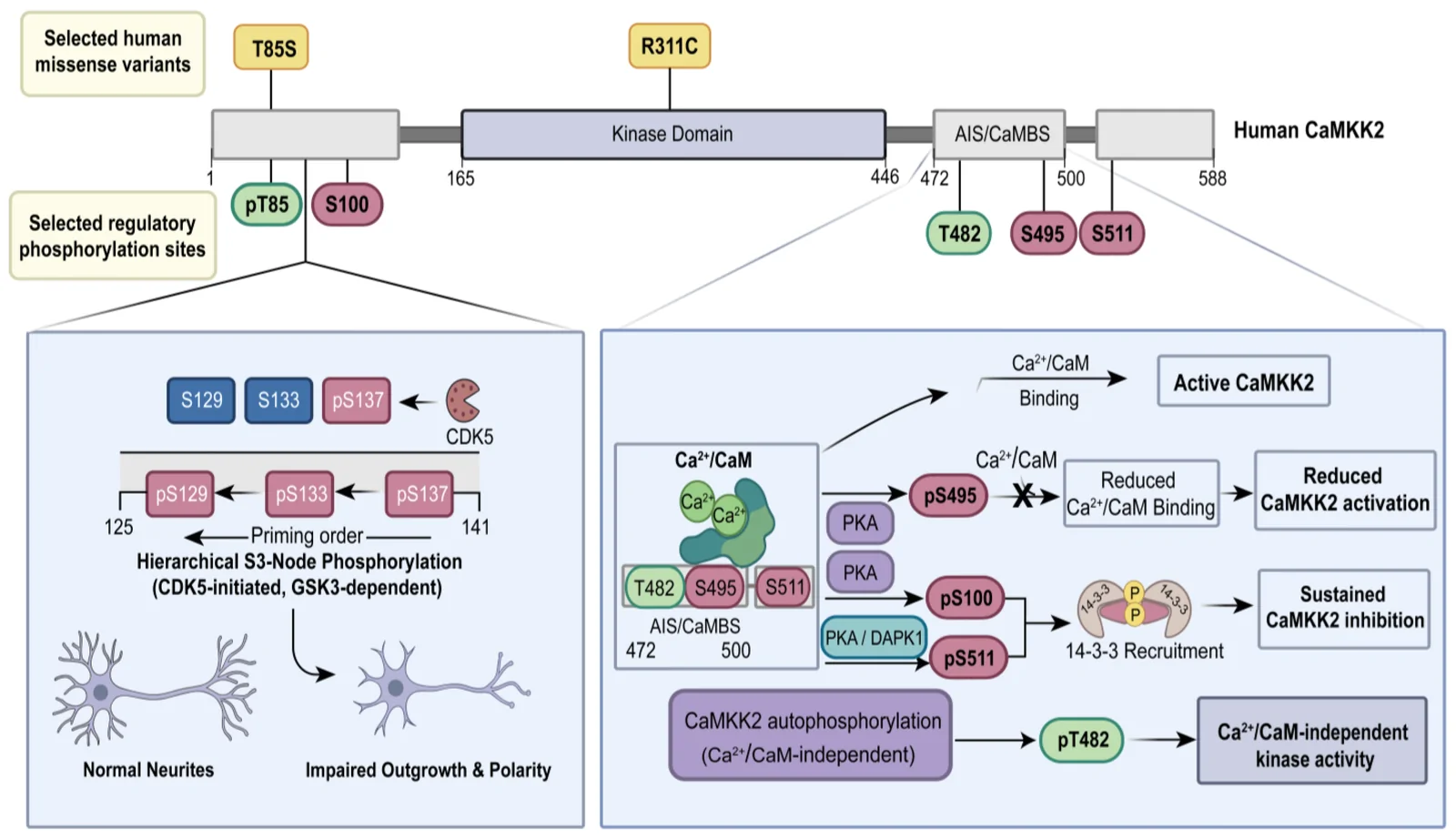
Domain organization and regulatory mechanisms of human CaMKK2. Schematic representation of human CaMKK2 showing its domain architecture, selected regulatory phosphorylation sites, and reported missense variants. The kinase domain and overlapping autoinhibitory/calmodulin-binding region (AIS/CaMBS) are indicated. Green and pink labels identify sites associated with activating autophosphorylation and inhibitory phosphorylation, respectively, whereas yellow labels indicate human missense variants. Within the S3 node, CDK5-mediated phosphorylation of Ser137 primes subsequent phosphorylation of Ser133 and Ser129 by GSK3, resulting in reduced CaMKK2 activity. This regulatory mechanism has been linked to impaired neurite outgrowth and neuronal polarity. Ca^2+^/CaM binding activates CaMKK2, whereas PKA-mediated phosphorylation of Ser495 reduces Ca^2+^/CaM binding and kinase activation. Phosphorylation of Ser100 and Ser511 promotes 14-3-3 recruitment and sustained inhibition of CaMKK2. DAPK1 has also been reported to phosphorylate Ser511. Ca^2+^/CaM-independent autophosphorylation at Thr482 contributes to autonomous kinase activity, whereas autophosphorylation at Thr85 supports sustained activation following transient Ca^2+^/CaM stimulation. The T85S variant disrupts this sustained activation mechanism, while R311C reduces catalytic activity and apparent Ca^2+^/CaM affinity. Although both variants have experimentally demonstrated effects on CaMKK2 function, their causal contributions to psychiatric disorders remain unestablished. The schematic summarizes reported regulatory mechanisms that may operate under different cellular conditions. Abbreviations: AIS, autoinhibitory sequence; CaM, calmodulin; CaMBS, calmodulin-binding sequence; CDK5, cyclin-dependent kinase 5; DAPK1, death-associated protein kinase 1; GSK3, glycogen synthase kinase 3; PKA, protein kinase A.

CaMKK2 is expressed in a wide range of human tissues, including the brain, liver, kidney, bone marrow, gastrointestinal tract, and reproductive organs [9]. Structurally, CaMKK2 exhibits a modular organization, consisting of a central kinase domain and a regulatory region that contains overlapping autoinhibitory sequence (AIS) and calmodulin-binding sequence (CaMBS), flanked by N- and C-terminal regions whose functions remain incompletely defined (Figure 1) [1].

In addition, CaMKK2 contains an arginine–proline-rich (RP) insert within the small N-lobe of the kinase domain, a feature shared with its paralogue CaMKK1. This region has been implicated in substrate recognition and may contribute to the activation of downstream targets, including CaMK1, CaMK4, and AMP-activated protein kinase (AMPK) [10].

The human *CAMKK2* gene gives rise to multiple transcript variants through alternative promoter usage and alternative splicing. A well-characterized full-length CaMKK2 isoform comprises 588 amino acids, whereas shorter isoforms differ in their C-terminal regions and kinase activity [11]. Unless otherwise indicated, this review refers to the full-length 588-amino-acid CaMKK2 isoform.

### 3.2. Mechanisms of CaMKK2

#### 3.2.1. Upstream Regulatory Mechanisms of CaMKK2 Activity

The activity of CaMKK2 is regulated through multiple mechanisms, including Ca^2+^/calmodulin-dependent activation, autophosphorylation, and phosphorylation at regulatory sites by upstream kinases such as protein kinase A (PKA) [6,8,12]. Binding of Ca^2+^-calmodulin to the calmodulin-binding sequence relieves autoinhibition by preventing the adjacent autoinhibitory region from interacting with the catalytic site, thereby promoting CaMKK2 activation [1]. This process is subject to inhibitory regulation by the PKA pathway. PKA phosphorylates Ser100, Ser495, and Ser511. Phosphorylation of Ser495 has been shown to reduce Ca^2+^/calmodulin binding, whereas phosphorylation of Ser100 and Ser511 promotes recruitment of 14-3-3 adaptor proteins, thereby limiting Ser495 dephosphorylation and contributing to sustained inhibition of CaMKK2 [13]. Death-associated protein kinase-1 (DAPK1) has also been reported to phosphorylate Ser511, a modification that may further modulate Ca^2+^/calmodulin-dependent activation [13,14]. A-kinase anchoring protein 11 (AKAP11) organizes PKA signaling within distinct cellular compartments and may indirectly influence CaMKK2 phosphorylation. However, a direct interaction between AKAP11 and CaMKK2 has not been demonstrated [13,15,16,17].

In addition to Ca^2+^-dependent regulation, CaMKK2 activity is influenced by autophosphorylation. Binding of Ca^2+^-calmodulin induces autophosphorylation at Thr85 within the N-terminal regulatory region, which has been proposed to support sustained activation following transient Ca^2+^ signals [6]. Autophosphorylation at Thr482 within the autoinhibitory region has also been associated with increased kinase activity [18].

CaMKK2 is further regulated by a Ca^2+^-independent mechanism involving sequential phosphorylation within the regulatory S3 node. Phosphorylation of Ser137 by cyclin-dependent kinase 5 (CDK5) primes subsequent phosphorylation of Ser133 and Ser129 by GSK3, resulting in inhibition of CaMKK2 activity [17]. This priming-dependent mechanism suggests a hierarchical mode of regulation. Experimental studies have shown that neurons expressing CaMKK2 variants that mimic constitutive phosphorylation at the S3 node exhibit impaired neurite outgrowth and disrupted axon-dendrite polarity, whereas variants that prevent phosphorylation at these sites display relatively normal neuronal morphology [6,17]. These findings indicate that S3 node-mediated regulation of CaMKK2 may contribute to the control of neuronal structure (Figure 1).

#### 3.2.2. Downstream Kinase Targets of CaMKK2 Signaling

**Figure 2:**
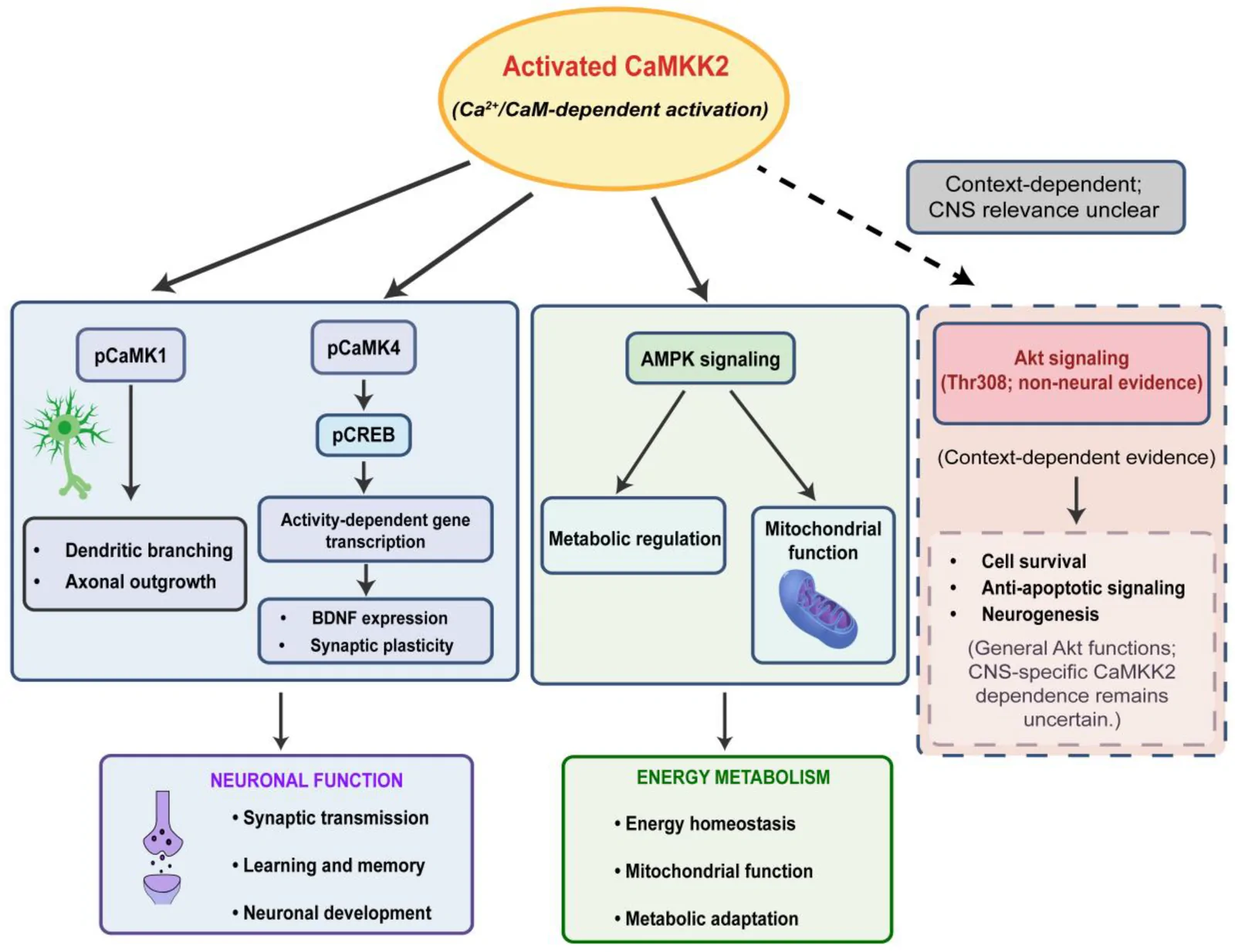
Major downstream signaling pathways and biological functions of activated CaMKK2. Ca^2+^/CaM activates CaMKK2, which in turn regulates CaMK1, CaMK4-CREB, and AMPK signaling. CaMK1 contributes to dendritic branching and axonal outgrowth, while CaMK4-CREB signaling regulates activity-dependent gene transcription, including BDNF expression, and supports neuronal development and synaptic plasticity. AMPK is involved in metabolic regulation, mitochondrial function, and cellular energy homeostasis, although these functions are not necessarily mediated by CaMKK2-dependent AMPK activation. Unlike the established CaMKK2 downstream targets CaMK1, CaMK4, and AMPK, the involvement of CaMKK2 in Akt regulation has not been established in CNS cells. Direct CaMKK2-mediated phosphorylation of Akt at Thr308 has been demonstrated in non-neural cancer cell models, whereas regulation of Akt Ser473 phosphorylation has been linked to an indirect mechanism involving mTORC2. The dashed arrow therefore indicates limited mechanistic validation of CaMKK2-Akt signaling in CNS cells rather than an exclusively indirect biochemical relationship. Abbreviations: Akt, protein kinase B; AMPK, AMP-activated protein kinase; BDNF, brain-derived neurotrophic factor; CaM, calmodulin; CREB, cAMP response element-binding protein; mTORC2, mechanistic target of rapamycin complex 2.

CaMKK2 acts as an upstream kinase that phosphorylates and activates several downstream targets, including CaMK1, CaMK4, and the α subunit of AMPK. Specifically, CaMKK2 phosphorylates conserved threonine residues within the activation loops of these kinases, such as Thr177 in CaMK1, Thr196 in CaMK4, and Thr172 in AMPK, thereby promoting their activation. In addition, CaMKK2 has also been reported to regulate Akt signaling in a context-dependent manner, although the mechanisms and their relevance to CNS cells remain incompletely defined. Through these downstream pathways, CaMKK2 contributes to the regulation of cellular processes including energy metabolism, synaptic plasticity, and neuronal function (Figure 2) [1,8,12,19,20,21,22].

Among these effectors, CaMK1 is involved in neuronal development and synaptic plasticity. It has been associated with processes such as axonal outgrowth, dendritic branching, and synapse formation [23,24,25,26,27]. In hippocampal neurons, CaMK1 is required for NMDA receptor-dependent long-term potentiation (LTP), a process underlying learning and memory [28]. Consistent with this, CaMKK2-deficient mice exhibit reduced LTP at hippocampal CA1 synapses, accompanied by impairments in long-term memory [5].

CaMK4, another downstream effector of CaMKK2, is involved in homeostatic plasticity, which helps stabilize neuronal activity within a physiological range through regulation of excitatory and inhibitory balance. CaMKK2-mediated phosphorylation is required for CaMK4 activation, and alterations in this signaling pathway have been associated with changes in neuronal excitability and activity homeostasis [29,30].

In addition to its role in synaptic regulation, CaMKK2 contributes to cellular energy homeostasis through activation of AMPK. However, AMPK can also be activated by LKB1 and other mechanisms. Changes in AMPK phosphorylation or downstream signaling alone are therefore insufficient to establish a role for CaMKK2 in a given cellular or disease context [2,31]. Neurons have high metabolic demands and limited glycogen reserves, relying on extracellular substrates supplied by astrocytes [32,33]. Under conditions of increased energy demand, AMPK promotes glucose uptake by facilitating translocation of the glucose transporter GLUT3 to the plasma membrane [34]. AMPK activation has also been associated with enhanced mitochondrial biogenesis and oxidative metabolism through regulation of factors such as peroxisome proliferator-activated receptor gamma coactivator-1α (PGC-1α) and mitochondrial transcription factor A (TFAM) [35,36]. CaMKK2 has been implicated in mitochondrial regulation in a cell type-specific manner, whereas AMPK-dependent pathways have been linked to mitochondrial redox homeostasis and PGC-1α-mediated metabolic adaptation across multiple experimental models [22,34,37,38,39,40].

CaMKK2 has also been implicated in the regulation of Akt/PKB signaling, a pathway involved in neuronal development and survival [41,42,43]. Unlike the established CaMKK2 substrates CaMK1, CaMK4, and AMPK, however, the evidence linking CaMKK2 to Akt regulation comes primarily from non-neuronal models. In cancer cells, CaMKK2 has been shown to directly phosphorylate Akt at Thr308, whereas its effects on Ser473 phosphorylation appear to involve indirect regulation through mTORC2 [44]. Whether CaMKK2-dependent Akt signaling operates in CNS cells or contributes to neurological disease remains unclear [45].

**Figure 3:**
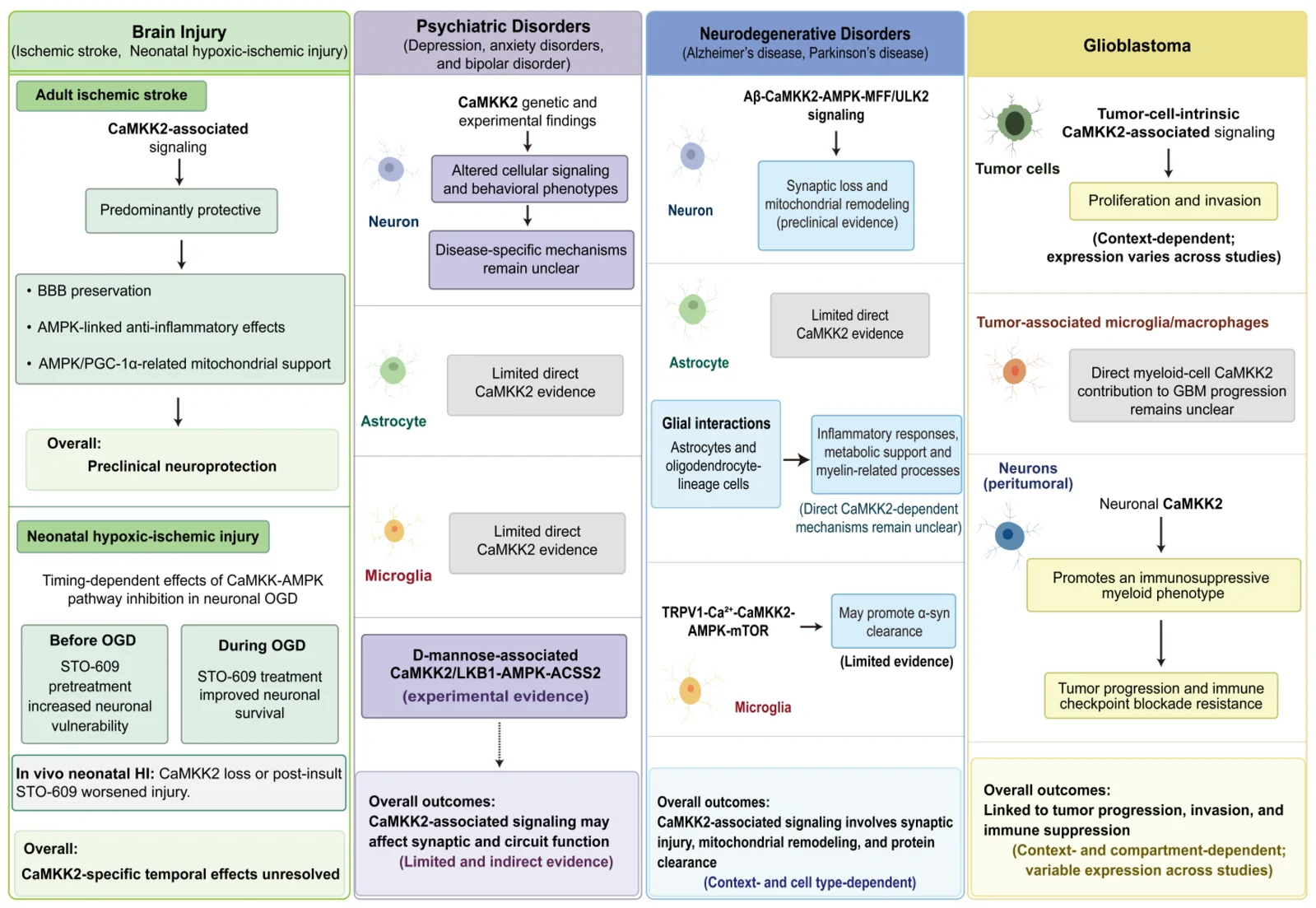
Context-dependent functions of CaMKK2-associated signaling across central nervous system disorders. Schematic overview of CaMKK2-associated signaling in brain injury, psychiatric disorders, neurodegenerative diseases, and glioblastoma. In adult ischemic stroke, preclinical evidence largely supports protective effects of CaMKK2-associated signaling. In neonatal hypoxic–ischemic injury, CaMKK2 deficiency or post-insult STO-609 treatment worsens injury, whereas the effects of STO-609 in neuronal oxygen-glucose deprivation models depend on intervention timing. Whether these timing-dependent effects are specific to CaMKK2 remains unclear. Evidence in psychiatric disorders comes from genetic, behavioral, and experimental signaling studies, although disease-specific mechanisms remain poorly defined. Among neurodegenerative disorders, the strongest experimental evidence supports neuronal Aβ-CaMKK2-AMPK signaling in AD. In PD, a targeted microglial TRPV1 intervention has been linked to CaMKK2-AMPK/mTOR signaling and α-synuclein clearance, although whether this effect depends specifically on CaMKK2 remains unclear. Astrocytes and oligodendrocyte-lineage cells, including NG2 glia, may also contribute to the regulation of inflammation, metabolic support, and myelin maintenance. However, direct evidence for CaMKK2-dependent mechanisms in these cell populations remains limited. In glioblastoma, CaMKK2-associated signaling in tumor cells has been linked to proliferation and invasion, while neuronal CaMKK2 has been implicated in immunosuppressive myeloid phenotypes and resistance to immune checkpoint blockade in experimental models. The figure highlights the context-dependent roles of CaMKK2-associated signaling and the varying strength of mechanistic evidence across these disorders. Abbreviations: Aβ, amyloid-β; AMPK, AMP-activated protein kinase; BBB, blood–brain barrier; mTOR, mechanistic target of rapamycin; PGC-1α, peroxisome proliferator-activated receptor gamma coactivator 1α; TRPV1, transient receptor potential vanilloid 1.

Collectively, CaMKK2 integrates signaling pathways that regulate synaptic function, energy metabolism, and cell survival, thereby contributing to neuronal homeostasis. Disruption of this network has been implicated in a range of central nervous system disorders. Notably, the functional consequences of CaMKK2 dysregulation appear to vary across disease contexts, reflecting its context-dependent roles in coordinating synaptic, metabolic, and survival pathways (Figure 3).

## 4. CaMKK2 and Brain Injury

### 4.1. CaMKK2 in Adult Ischemic Stroke

Ischemic stroke results from reduced cerebral blood flow, leading to hypoxia, metabolic failure, and subsequent neuronal injury. These processes are characterized by disruptions in energy homeostasis and inflammatory signaling, pathways in which CaMKK2-dependent mechanisms have been implicated.

Research in animal models of cerebral ischemia–reperfusion injury (CIRI) has shown that inhibition of CaMKK2 exacerbates ischemia-induced apoptotic pathways in brain endothelial cells, resulting in reduced endothelial cell viability, enhanced inflammatory responses, and increased blood–brain barrier (BBB) disruption following ischemia. Conversely, maintenance of CaMKK2 signaling has been associated with preserved endothelial function, reduced leukocyte adhesion, and improved BBB integrity [46]. Consistent with these findings, genetic deletion of CaMKK2 worsens post-stroke outcomes, including infarction, hemorrhagic transformation, and cerebral edema [47]. AMPK represents another downstream pathway through which CaMKK2 may regulate responses to cerebral ischemia. CaMKK2 activates AMPK by directly phosphorylating it at Thr172 [48]. In experimental models of neuroinflammation, exogenous H_2_S has been reported to suppress inflammatory activation through CaMKK2-dependent AMPK signaling [49]. Similarly, in cerebral ischemia, reduced endogenous CBS-H_2_S signaling has been linked to decreased AMPK activation and increased microglial inflammation. Pharmacological inhibition of CaMKK2 also weakened the anti-inflammatory effects of H_2_S, suggesting that CaMKK2 contributes to this response [50]. Although some of the cited studies used the M1/M2 classification [49,50], this classification does not fully capture the diversity of microglial inflammatory responses. Microglia exhibit diverse and dynamic phenotypes that change with local environmental conditions and injury stage [51]. CaMKK2 knockdown experiments support a role for CaMKK2 in H_2_S-induced AMPK activation and anti-inflammatory responses in microglia [49]. Although AMPK has been linked to the suppression of NF-κB-associated inflammatory signaling in other experimental settings [52], direct evidence that CaMKK2 regulates NF-κB signaling or inflammasome activation following cerebral ischemia remains limited.

CaMKK2-associated signaling has also been linked to mitochondrial function and oxidative stress. Mild hypothermia has been shown to reduce ischemic injury, accompanied by increased CaMKK2-AMPK signaling and decreased S100A8 expression. In the same study, the CaMKK inhibitor STO-609 weakened the protective effect of hypothermia. However, this pharmacological evidence alone is insufficient to establish that the effect is specifically mediated by CaMKK2 [53]. In a separate study, compound 3C reduced infarct volume and improved neurological outcomes, alongside activation of CaMKK2/AMPK/PGC-1α signaling [54]. CaMKK2 inhibition also weakened the anti-inflammatory effects of compound 3C in microglial cultures, although the specific contribution of CaMKK2 to neuroprotection in vivo remains unclear.

Additional studies have linked CaMKK2-associated signaling to the neuroprotective effects of specific interventions in cerebral ischemia/reperfusion injury. Xu et al. reported that stanniocalcin-1 (STC-1) interacted with calreticulin and was associated with activation of the CaMKK2/AMPK/GSK-3β/Nrf2 signaling axis in a mouse model of cerebral ischemia/reperfusion injury. Intranasal STC-1 administration improved neurological outcomes and reduced infarct volume, blood–brain barrier permeability, neuronal apoptosis, oxidative stress, and ferroptosis-related changes. These protective effects were abolished by AMPK inhibition [55]. In a separate study, Liu et al. identified *CAMKK2* as a target of miR-21-3p and showed that miR-21-3p overexpression attenuated the neuroprotective effects of OL-FS13 in oxygen-glucose deprivation (OGD)/reoxygenation-treated PC12 cells and rats subjected to cerebral ischemia/reperfusion. Treatment with the CaMKK inhibitor STO-609 also weakened OL-FS13-associated AMPK activation and Nrf2 upregulation, implicating the miR-21-3p/CaMKK2/AMPK/Nrf2 axis in these experimental models. Together, these studies suggest that CaMKK2-associated signaling contributes to the neuroprotective effects of STC-1 and OL-FS13, although the evidence for a specific role of CaMKK2 differs between the two interventions [55,56].

Genetic deletion and pharmacological inhibition studies support a protective role for CaMKK2-associated signaling after adult ischemic stroke, particularly in neurovascular injury [46,47]. However, the specific contribution of CaMKK2 to several AMPK-associated metabolic and inflammatory responses remains unclear [49,50,54,55,56]. These findings should also be considered within the broader neurovascular unit, where endothelial cells, pericytes, astrocytic endfeet, neurons, and microglia interact to maintain vascular integrity, provide metabolic support, and regulate inflammation after ischemia [57]. Whether CaMKK2 regulates these interactions, particularly through its actions in pericytes or astrocytes, remains to be determined.

### 4.2. CaMKK2 in Neonatal Hypoxic–Ischemic Injury

Neonatal hypoxic–ischemic (HI) injury results from reduced cerebral blood flow and oxygen deprivation during the perinatal period, leading to metabolic failure and subsequent neuronal injury. These pathological processes involve disruptions in energy homeostasis and inflammatory responses [58,59]. In this context, CaMKK2-dependent signaling has been implicated in experimental neonatal hypoxic–ischemic injury [60].

Studies of neonatal HI have reported differing effects of CaMKK2-associated signaling. Min et al. [60] found that CaMKK2 deletion increased infarct volume in postnatal day 10 mice subjected to HI. Treatment with the CaMKK inhibitor STO-609 shortly after HI also increased infarct volume at 24 h and worsened tissue loss and functional recovery at three weeks. STO-609 treatment was associated with reduced levels of phosphorylated CaMK4, Bcl-2, collagen IV, and claudin-5, along with increased Bax expression. These results support a protective role for CaMKK2-associated signaling, although the effects of STO-609 cannot be attributed solely to CaMKK2. Rousset et al. [61] investigated AMPK activation in postnatal day 9 mice subjected to HI and in embryonic cortical neurons subjected to OGD. Experiments using CaMKK2-deficient neurons showed that sustained AMPK activation during OGD involved CaMKK2 and predominantly AMPKα1-containing complexes. In neuronal cultures, STO-609 treatment during OGD reduced AMPK activation and cell death, whereas pretreatment followed by a 3 h recovery period increased neuronal vulnerability. A similar trend was observed with a 24 h recovery interval, but the effect did not reach statistical significance. Whereas Min et al. assessed brain injury and functional outcomes in vivo, the timing-dependent effects reported by Rousset et al. were observed in isolated neurons, with cell death assessed primarily by LDH release. These differences complicate direct comparisons between the studies and do not establish opposing, time-dependent functions of CaMKK2.

Beyond its effects on apoptosis, CaMKK2-associated signaling may also influence pathways involved in oxidative stress regulation and metabolic adaptation. One proposed mechanism involves engagement of the AMPK-Nrf2 axis, which may enhance antioxidant defense through induction of genes such as HO-1 and NQO1. However, this mechanism is mainly inferred from studies of ischemic and oxidative stress models, and whether CaMKK2 directly engages AMPK-Nrf2 signaling in neonatal HI remains to be determined [62,63,64,65]. Similarly, whether CaMKK2-associated signaling directly regulates NF-κB activity or inflammasome activation in neonatal HI remains unclear.

The differing findings of Min et al. and Rousset et al. may reflect differences between their in vivo and neuronal OGD models rather than opposing functions of CaMKK2. In particular, the timing-dependent effects of STO-609 observed in neuronal cultures have not been demonstrated with CaMKK2-specific interventions in vivo. Further studies using temporally controlled CaMKK2 manipulation and AMPK isoform-specific analyses are needed to determine how intervention timing, cellular context, and downstream signaling contribute to these differences.

## 5. CaMKK2 in Psychiatric Disorders

### 5.1. CaMKK2 in Depression, Anxiety, and Schizophrenia

Depression and anxiety disorders are characterized by disturbances in emotional regulation, cognitive function, and stress responsivity, accompanied by alterations in neural circuit activity and neuroendocrine signaling [66,67]. Their pathogenesis is multifactorial, involving genetic susceptibility, immune dysregulation, and metabolic imbalance [68,69]. Despite extensive investigation, the absence of reliable objective biomarkers continues to constrain diagnostic precision.

A recent study found that D-mannose activates AMPK through both Ca^2+^-CaMKK2 and lysosomal axis inhibition protein (AXIN)-liver kinase B1 (LKB1) signaling, thereby promoting ACSS2 nuclear translocation and TPH2 transcription. D-mannose treatment also increased BDNF levels and reduced eEF2 phosphorylation, alongside antidepressant-like effects in experimental models [70]. Because AMPK can be activated through multiple upstream pathways in this model, the specific contribution of CaMKK2 to these molecular and behavioral changes remains unclear. Thus, the findings suggest a possible role for CaMKK2-associated signaling in antidepressant-like responses but do not establish CaMKK2 as a causal factor in major depressive disorder.

Evidence linking CaMKK2 to anxiety-related phenotypes comes mainly from genetic association studies and animal models. The intronic variant rs1063843 has been associated with changes in CAMKK2 expression in the dorsolateral prefrontal cortex [71,72], but its direct effects on CaMKK2 kinase activity remain unknown. In contrast, the exonic variant rs3817190, which encodes the T85S substitution, has been linked to anxiety-related traits and experimentally shown to impair sustained autonomous activation of CaMKK2 [6]. However, neither variant has been established as a causal factor in human psychiatric disorders. CaMKK2-deficient mice showed reduced exploration of the center of the open field and spent less time in the open areas of the elevated zero maze, findings interpreted as anxiety-like behavior [6]. These mice also exhibited increased locomotor activity, which may complicate the interpretation of exploration-based measures. Thus, although the behavioral findings suggest that CaMKK2 may be involved in anxiety-related responses, they do not establish a causal link to human anxiety disorders.

In depression, it remains unclear whether CaMKK2 is required for D-mannose-associated AMPK-ACSS2 signaling and how its contribution differs from that of the parallel LKB1 pathway [70]. In studies of anxiety-related behavior, increased locomotor activity in CaMKK2-deficient mice may complicate the interpretation of exploration-based tests [6]. Further studies are needed to distinguish CaMKK2-dependent effects from those mediated by parallel signaling pathways and to determine whether the observed behavioral changes reflect anxiety-related responses or altered locomotor activity.

Schizophrenia has also been associated with CaMKK2. Luo et al. reported reduced *CAMKK2* expression in individuals with schizophrenia and identified genetic associations between *CAMKK2* variation and disease susceptibility [72]. Although these findings support a potential association, they do not establish a causal role for CaMKK2 or define the underlying disease mechanisms. Direct mechanistic evidence in schizophrenia remains limited.

### 5.2. CaMKK2 in Bipolar Disorder

Bipolar disorder is a chronic psychiatric condition characterized by recurrent manic and depressive episodes, affecting over 1% of the global population [73]. Its pathophysiology involves dysregulation of calcium-dependent signaling, synaptic plasticity, and cellular energy metabolism within corticolimbic circuits [74]. These convergent processes implicate CaMKK2-associated signaling as a potential regulatory node.

Lithium inhibits GSK3, which phosphorylates the CaMKK2 S3 regulatory node and suppresses CaMKK2 activity [17]. This suggests a possible molecular link between lithium treatment and CaMKK2 regulation, but does not establish that CaMKK2 mediates lithium’s therapeutic effects in bipolar disorder. Lithium has also been reported to increase CaMKK2 expression in the striatum, although the significance of this change and its relationship to treatment response remain unclear [3].

Genetic evidence linking CaMKK2 to bipolar disorder remains limited and heterogeneous. The rare R311C missense variant, identified de novo in an individual with bipolar disorder, has been shown to markedly impair CaMKK2 catalytic activity and Ca^2+^/CaM-dependent activation [75]. However, these biochemical findings do not establish R311C as a confirmed disease-causing variant. Although genome-wide association studies have not established *CAMKK2* as a robust susceptibility locus, case–control studies have reported nominal associations, including one involving the rs1063843 T allele. This allele has also been linked to reduced CaMKK2 expression in postmortem brain tissue [71,72,76]. These findings point to a possible contribution of CaMKK2 to bipolar disorder susceptibility but do not establish it as a primary causal driver.

CaMKK2-deficient mice have shown increased locomotor activity in the open-field test, reduced immobility in the tail-suspension test, impaired prepulse inhibition (PPI) of the acoustic startle response, and enhanced cued fear conditioning and fear-potentiated startle responses [3,6]. These findings indicate changes in several behavioral domains rather than a phenotype specific to any single psychiatric disorder. Reduced immobility in the tail-suspension test should be interpreted with caution, given the increased locomotor activity observed in these mice. Impaired PPI suggests altered sensorimotor gating but was also accompanied by increased baseline startle responses. Enhanced fear conditioning and fear-potentiated startle responses may reflect changes in fear processing, but these findings do not establish that CaMKK2-deficient mice model bipolar disorder or other human psychiatric conditions. The neural circuits involved and their relevance to specific psychiatric disorders remain unclear.

CaMKK2 regulates pathways involved in neuronal plasticity and metabolic homeostasis, including CaMK1/CaMK4 and AMPK signaling. Although these pathways are relevant to processes implicated in bipolar disorder, their roles in disease pathophysiology and the extent to which their dysregulation reflects altered CaMKK2 activity remain unclear [22,26,77]. Altered CaMKK2 activity has also been suggested to contribute to mitochondrial and redox dysfunction, but direct evidence supporting this link in bipolar disorder is still limited [3,22].

In bipolar disorder, it remains unclear whether functionally characterized *CAMKK2* variants contribute to disease-related neuronal changes or whether CaMKK2 is involved in responses to lithium treatment [3,17,75,76]. Behavioral changes observed in CaMKK2-deficient mice are not specific to bipolar disorder, and the proposed links between CaMKK2 and AMPK- or redox-related pathways have yet to be adequately validated in this disease [3,6,22,77].

## 6. CaMKK2 and Neurodegenerative Diseases

### 6.1. CaMKK2 in Alzheimer’s Disease

Alzheimer’s disease (AD), the most common form of dementia, is characterized by extracellular amyloid plaques containing amyloid-β (Aβ) peptides and intracellular neurofibrillary tangles composed of hyperphosphorylated Tau. Aβ peptides are produced through sequential cleavage of amyloid precursor protein (APP) by β-secretase (BACE1) and γ-secretase [78]. Aβ oligomers trigger NMDA receptor-dependent Ca^2+^ influx, leading to activation of CaMKK2-AMPK signaling. Direct experimental evidence links this pathway to Tau phosphorylation, synaptic dysfunction, and alterations in mitochondrial dynamics, including fission and mitophagy [79,80]. These findings support the view that CaMKK2 integrates excitotoxic and metabolic stress signals during Aβ-induced neurotoxicity rather than acting as an independent upstream driver of disease pathology.

Direct evidence implicates CaMKK2-AMPK signaling primarily in Aβ-induced synaptic dysfunction and Tau phosphorylation [80]. Beyond synaptic dysfunction and Tau phosphorylation, AMPK has also been implicated in the regulation of BACE1 expression in experimental models [81]. The specific contribution of CaMKK2 to individual inflammatory pathways in AD remains poorly defined. In particular, further investigation is needed to determine whether CaMKK2-dependent AMPK signaling modulates NF-κB activity, inflammasome activation, or oxidative stress-related inflammatory responses in AD, and whether these effects vary between neurons and glial cells. Postmortem analyses have reported reduced CaMKK2, transferrin, and transferrin receptor protein levels alongside increased cortical iron content in AD brains, although CaMKK2 abundance was not significantly correlated with iron content [82]. Regulation by non-coding RNAs, including miR-9-mediated suppression of CaMKK2, further supports its involvement in Aβ-associated synaptic dysfunction [83].

Experimental studies have linked Aβ-induced CaMKK2-AMPK activation to synaptic loss, dendritic spine alterations, and mitochondrial dysfunction [79,80]. More specifically, in an Aβ42 oligomer model, AMPK-dependent phosphorylation of mitochondrial fission factor (MFF) and Unc-51-like autophagy activating kinase 2 (ULK2) was linked to mitochondrial fission and mitophagy, respectively. Functional perturbation of these effectors attenuated Aβ42-induced synaptic loss, linking mitochondrial remodeling to synaptic dysfunction [79]. Additional studies have linked Aβ-induced metabolic stress to AMPK-mTOR-JNK3 signaling [84], whereas RAGE-associated CaMKK2-AMPK signaling has also been linked to Aβ-induced autophagosome formation in SH-SY5Y cells, as shown by pharmacological and siRNA-based experiments [85]. However, whether CaMKK2 regulates autophagic flux or pathological protein clearance in vivo remains unclear. Separately, miR-9 has been reported to target CaMKK2 and attenuate Aβ-induced synaptotoxicity, providing more direct evidence of a regulatory relationship between miR-9 and CaMKK2 in this experimental setting [83,86].

Although current evidence mainly links CaMKK2-AMPK activation to Aβ-induced synaptic dysfunction, the role of CaMKK2 in AD may extend beyond this process [79,80]. In postmortem AD cortex, reduced CaMKK2 abundance has been reported alongside lower levels of transferrin and transferrin receptor, although a direct relationship between CaMKK2 abundance and cortical iron content was not demonstrated [82]. These findings suggest that CaMKK2 dysregulation may be relevant to cellular processes beyond Aβ-related synaptotoxicity, but they do not establish a protective role for CaMKK2. Thus, while CaMKK2-AMPK signaling has been linked to detrimental effects in specific Aβ-related experimental models, opposing roles of CaMKK2 in AD have not yet been established through independently validated mechanisms.

In AD, experimental studies have linked CaMKK2-AMPK signaling to Aβ-induced synaptic dysfunction, Tau phosphorylation, and mitochondrial remodeling [79,80]. However, whether CaMKK2 regulates autophagic flux or inflammatory signaling in disease-relevant cell populations remains unclear. The relationship between reduced CaMKK2 abundance in postmortem AD cortex and findings from experimental models also requires further investigation [82,85].

### 6.2. CaMKK2 in Parkinson’s Disease

Parkinson’s disease (PD) is characterized by progressive neuronal loss and accumulation of α-synuclein aggregates that propagate in a prion-like manner and drive neuronal dysfunction [87,88]. At the cellular level, α-syn pathology engages mitochondrial dysfunction, oxidative stress, and impaired proteostasis, which progressively compromise neuronal homeostasis [87,89]. These alterations are closely linked to metabolic regulation and protein clearance pathways, providing a rationale for examining CaMKK2-associated signaling in PD.

Microglia are the principal immune cells of the CNS and participate in the clearance of pathological protein aggregates, including α-synuclein [90]. Under disease conditions, however, phagocytic and degradative functions may become compromised, limiting efficient α-syn clearance [89,91]. TRPV1-mediated Ca^2+^ influx has been implicated in neuroimmune regulation [92]. In an experimental model of PD, targeted photothermal activation of microglial TRPV1 channels promoted α-synuclein clearance alongside activation of Ca^2+^-CaMKK2-AMPK-mTOR signaling [93]. However, these findings are limited to a specific intervention model and do not establish whether CaMKK2 plays a broader role in α-synuclein pathology or directly regulates NF-κB signaling, inflammasome activation, or other inflammatory processes in PD. Although enhanced microglial autophagy and α-synuclein clearance have been reported, the specific contribution of CaMKK2 and the extent to which these findings reflect changes in autophagic flux remain to be clarified.

Beyond the microglial intervention study, transcriptomic and bioinformatic analyses have identified *CAMKK2* as a candidate gene in PD-associated molecular networks [94]. These analyses have also suggested links to AMPK-related energy regulation and autophagy, as well as predicted interactions with differentially expressed microRNAs, including miR-30e-5p [94]. However, these findings remain hypothesis-generating and do not establish that *CAMKK2* functionally regulates these pathways in PD or that the predicted microRNA interactions occur in disease-relevant cells. Experimental studies are needed to determine whether these associations reflect functional mechanisms.

In PD, it remains unclear whether CaMKK2 is directly involved in the microglial clearance of α-synuclein observed following TRPV1-targeted intervention [93]. Studies using CaMKK2-specific manipulation and direct assessments of autophagic degradation in independent PD models are needed to clarify its role. Transcriptomic and bioinformatic findings alone cannot establish a causal relationship between CaMKK2 and α-synuclein clearance [94].

Evidence linking CaMKK2 to neurodegeneration is stronger in AD than in PD. In AD, experimental studies have linked CaMKK2-AMPK signaling to Aβ-associated synaptic dysfunction, Tau phosphorylation, and mitochondrial alterations, whereas evidence in PD remains limited, relying mainly on a specific intervention model and transcriptomic or bioinformatic analyses. In both disorders, the specific contribution of CaMKK2 to autophagic degradation and pathological protein clearance remains unclear. Beyond microglia, astrocytes and oligodendrocyte-lineage cells, including NG2 glia (oligodendrocyte precursor cells), have been implicated in neurodegenerative processes through their involvement in neuron-glia interactions, myelin homeostasis, and inflammatory responses [95,96]. Changes in astrocytic metabolic support, oligodendroglial myelin maintenance, and microglial inflammatory activity may influence the context in which CaMKK2-associated signaling operates, with potential effects on neuronal energy balance and synaptic vulnerability. Whether CaMKK2 signaling in specific neuronal or glial populations directly regulates these cellular interactions in AD or PD, however, remains unclear. Although CaMKK2 knockdown studies support its involvement in H_2_S-induced anti-inflammatory responses in microglial models [49], these findings do not establish equivalent CaMKK2-dependent mechanisms in AD or PD. Cell type-specific manipulation of CaMKK2 is needed to determine whether inflammatory responses and pathological protein clearance in these disorders are directly regulated by CaMKK2 rather than merely associated with downstream signaling pathways.

## 7. CaMKK2 and Glioblastoma

Glioblastoma (GBM) is an aggressive primary brain tumor characterized by rapid progression, diffuse infiltration, and poor clinical outcomes despite current therapies [97,98,99]. Hallmark features, including pronounced angiogenesis, hypoxia, and necrosis, reflect a highly adaptive and invasive phenotype [100,101]. These processes are closely linked to tumor cell proliferation, invasion, and dynamic interactions with the surrounding microenvironment, providing a framework for examining CaMKK2-associated signaling in GBM.

Clinical and experimental studies have reported elevated *CAMKK2* expression in high-grade gliomas. Promoter methylation has also been linked to *CAMKK2* expression, and higher expression has been associated with poorer prognosis and aggressive tumor features, including migration, invasion, and proliferation [102]. The tumor-suppressive microRNA miR-1271, which is downregulated in GBM, has been reported to directly target the 3′-UTR of *CAMKK2* mRNA. Restoring miR-1271 expression reduces CaMKK2 levels, Akt phosphorylation, and Ki-67 expression, consistent with reduced tumor cell proliferation [103]. However, these findings do not establish that the effects of miR-1271 are mediated exclusively by CaMKK2. Conversely, lower *CAMKK2* expression has been reported in canine gliomas and in some human glioma datasets, including comparisons of GBM tissue and patient-derived GBM cell lines with non-tumor brain tissue [104]. These findings do not necessarily conflict with the reported association between higher *CAMKK2* expression and higher glioma grade [102], as the studies used different cohorts and comparison groups. *CAMKK2* expression also increased following induced differentiation of patient-derived GBM cells, suggesting that it may vary with tumor cell state [104]. Whether the divergent expression patterns reflect differences in tumor grade, cellular composition, differentiation state, or other biological and methodological factors remains unclear.

In vivo studies provide functional evidence that host CaMKK2 contributes to the immunosuppressive microenvironment in GBM [105]. In experimental GBM models, conditional deletion of neuronal CaMKK2 increased MHC-II expression in tumor-associated myeloid cells, prolonged survival, and enhanced responses to immune checkpoint blockade (ICB). Bone marrow chimera experiments further indicated that CaMKK2 deficiency in non-hematopoietic host cells was required for improved survival, enhanced ICB responses, and the emergence of an MHC-II-high tumor-associated myeloid phenotype. Combined deficiency in non-hematopoietic and hematopoietic compartments produced additional effects on survival and antitumor T-cell phenotypes, including changes in CD4^+^ tumor-infiltrating lymphocytes and reduced CD8^+^ T-cell exhaustion [105]. These findings support a functional role for host CaMKK2 in immune suppression in the models studied. However, the contributions of individual cellular compartments and the relevance of these findings to human GBM remain to be established.

In GBM, studies of tumor cells and genetic manipulation of host cells suggest that CaMKK2 may have different roles depending on the cellular context [103,105]. However, the relative contributions of neuronal, myeloid, and tumor cells remain unclear. Further work is also needed to explain differences in CAMKK2 expression across patient cohorts and tumor-cell states and to determine whether findings from experimental models are relevant to human GBM [102,103,104,105].

Table 1 compares the principal findings, evidence types, and unresolved questions across the CNS disorders reviewed.

## 8. Therapeutic Targeting of CaMKK2 in CNS Disorders

### 8.1. Current Pharmacological Tools and Their Limitations

The pharmacological development of CaMKK2 inhibitors has moved from widely used but imperfect tool compounds toward more selective chemical probes. Nevertheless, current inhibitors remain constrained by off-target effects, limited selectivity between CaMKK1 and CaMKK2, and inadequate pharmacokinetic characterization for CNS applications.

STO-609 remains the most widely used CaMKK2 inhibitor and has been extensively employed in cellular and animal studies [21,106]. Although initially considered relatively selective for CaMKK1/2, subsequent kinome profiling identified several off-target kinases, including casein kinase 2 (CK2), MAP kinase-interacting serine/threonine kinase 1 (MNK1), PIM serine/threonine kinase 3 (PIM3), and extracellular signal-regulated kinase 8 (ERK8). These findings indicate that phenotypes observed with STO-609 should not be attributed exclusively to CaMKK2 inhibition without complementary genetic or orthogonal pharmacological validation [107,108]. GSK650394 exhibits substantially higher biochemical potency than STO-609 but poorer kinase selectivity. In a 334-kinase screen, 29 kinases were inhibited by more than 90% at 1 μM, demonstrating its broad off-target activity [106,108]. This limited selectivity complicates the attribution of downstream signaling changes to CaMKK2 inhibition alone. Nevertheless, GSK650394 has served as a starting point for subsequent medicinal chemistry efforts to improve CaMKK2 inhibitor selectivity.

Compound 4t represented an important step toward CNS-directed CaMKK2 pharmacology. This 2,4-diaryl 7-azaindole derivative showed single-digit nanomolar potency, oral bioavailability, and brain penetration. In a hypothalamus-derived cell model, 4t inhibited AMPK phosphorylation, and oral administration reduced ghrelin-induced food intake in rodents [109]. These findings support the feasibility of brain-penetrant pharmacological modulation consistent with CaMKK2 inhibition, although selective target engagement and therapeutic efficacy in CNS disease models remain to be established. The kinase selectivity and pharmacokinetic properties of compound 4t also remain only partially optimized [106,109].

More recent medicinal chemistry efforts have focused on improving selectivity. A hinge-binding scaffold-hopping strategy yielded compounds 13g and 45 with improved selectivity for CaMKK2 across the kinome compared with earlier inhibitors [106]. SGC-CAMKK2-1 represents a further advance, with an enzymatic IC_50_ of approximately 30 nM, substantially improved kinome selectivity, and demonstrated cellular target engagement by NanoBRET. The structurally related analogue SGC-CAMKK2-1N serves as a negative control in cellular assays to help distinguish compound-specific effects [110]. However, SGC-CAMKK2-1 retains activity against CaMKK1, and preliminary in vivo pharmacokinetic data led the authors to recommend its use primarily in cell-based assays [110].

Table 2 compares the pharmacological properties of representative CaMKK2 inhibitors. Despite improvements in biochemical potency and selectivity, selective CaMKK2 target engagement and therapeutic efficacy in CNS disease models remain to be established.

### 8.2. CNS Exposure and Target Engagement

Brain penetration is an important but insufficient criterion for evaluating the therapeutic potential of CaMKK2 inhibitors. Although compound 4t has been reported to cross the blood–brain barrier and exert pharmacological effects after oral administration [109], available evidence remains insufficient to establish adequate CaMKK2 target engagement in relevant CNS cell types [110]. Total brain concentrations alone may not reflect the unbound drug concentration available for intracellular target interaction. Future studies should therefore integrate plasma and brain pharmacokinetics with protein binding and unbound brain exposure to determine whether concentrations sufficient for target engagement are achieved.

Target engagement represents a further challenge. SGC-CAMKK2-1 provided an important advance by demonstrating cellular target engagement using NanoBRET, although such evidence has not yet been established in relevant CNS tissues or disease models [110]. Moreover, changes in downstream biomarkers such as AMPK phosphorylation cannot by themselves establish direct CaMKK2 engagement, as these pathways are regulated by multiple upstream kinases and are influenced by cellular state. Direct target-engagement measurements should therefore be combined with pharmacodynamic biomarkers and orthogonal genetic validation.

### 8.3. Context-Dependent Liabilities of CaMKK2 Modulation

The context-dependent functions of CaMKK2 pose an additional challenge for therapeutic targeting. In adult ischemic stroke, CaMKK2 has been associated with endogenous neuroprotective responses, including maintenance of blood–brain barrier integrity. Recent studies have further linked the CaMKK2-AMPK pathway to antioxidant and cytoprotective responses following cerebral ischemia/reperfusion [47,55,56]. CaMKK2 inhibition could therefore interfere with endogenous protective mechanisms. In neonatal hypoxic–ischemic injury, experimental studies have shown that the effects of CaMKK-AMPK pathway inhibition may depend on intervention timing, while the temporal effects of CaMKK2-specific inhibition remain unclear [60,61]. These findings highlight the time-dependent effects of AMPK-associated signaling in injury but do not establish a similar temporal pattern for CaMKK2-specific inhibition. Developmental stage, the duration of pathway activation, and cellular metabolic state may also shape the response to pathway modulation, although their specific contributions to CaMKK2-dependent effects remain unclear.

Similar context dependence is evident across neurodegenerative disease and glioblastoma. In Alzheimer’s disease (AD), aberrant CaMKK2-AMPK signaling has been implicated in Aβ-induced synaptic loss and mitochondrial dysfunction. In experimental glioblastoma (GBM) models, host CaMKK2, particularly neuronal CaMKK2, has been shown to contribute to an immunosuppressive tumor microenvironment and resistance to immune checkpoint blockade [79,80,105]. Thus, how CaMKK2 is targeted may need to be considered in relation to both the disease context and the cellular compartment involved.

Comparisons across these disease contexts suggest that the effects of CaMKK2-associated signaling depend on cellular context and downstream pathway engagement. In cerebral ischemia, experimental studies support protective roles for CaMKK2 in maintaining endothelial integrity and regulating microglial inflammation, together with AMPK-associated metabolic responses that may help cells adapt to energy stress [46,47,49,50]. In contrast, CaMKK2-AMPK activation in Aβ-exposed neurons has been linked to Tau phosphorylation and MFF/ULK2-dependent mitochondrial remodeling, contributing to synaptic loss [79,80]. In GBM, neuronal CaMKK2 influences tumor-associated myeloid phenotypes and responses to immune checkpoint blockade, while studies in tumor cells have linked CaMKK2 to proliferation and invasion [102,103,105]. These findings point to cell type and downstream pathway engagement as important factors shaping the effects of CaMKK2 signaling. Intracellular Ca^2+^ dynamics, metabolic state, and disease stage may also contribute to these differences, although direct comparative evidence for their roles remains limited.

These differences argue against uniform CaMKK2 inhibition across CNS disorders. Therapeutic strategies should consider both the pathological effects of CaMKK2 in the intended cellular compartment and the risk of disrupting protective functions elsewhere.

### 8.4. Translational Priorities

Translating CaMKK2 inhibition into CNS therapeutics will require more than improving biochemical potency. Selectivity for CaMKK2 over CaMKK1 and across the broader kinome remains important because several commonly used inhibitors have substantial off-target activity [108,110]. CNS studies should also determine whether sufficient unbound drug reaches the brain and whether CaMKK2 is engaged at these concentrations, rather than using BBB penetration as a surrogate for target exposure [111,112]. Combining pharmacological inhibitors with genetic approaches and direct cellular assays, such as NanoBRET, can help distinguish CaMKK2-dependent effects from those caused by off-target activity [106,110]. Where feasible, re-expression of wild-type CaMKK2 following genetic loss of function, with kinase-inactive CaMKK2 as a control, would provide stronger evidence for a causal role. In pharmacological studies, inhibitor-resistant rescue or the use of structurally distinct inhibitors could help distinguish on-target effects from off-target activity.

Pharmacodynamic studies also need to consider cellular and pathological context, particularly when CaMKK2-associated signaling may have different effects under different conditions [61,105]. More specific biomarkers of CaMKK2 target engagement are needed, as changes in AMPK phosphorylation alone cannot establish CaMKK2 engagement because AMPK can be activated by multiple upstream kinases [31,110]. Future studies should combine cell type-specific conditional knockout models and temporally controlled CaMKK2 manipulation with single-cell or single-nucleus RNA sequencing and spatial transcriptomics to define molecular changes across cell types and disease stages and determine their functional significance. Cell-type-directed drug delivery, combined with measurements of cellular drug exposure and target engagement, may also help determine whether pharmacological effects are attributable to CaMKK2 modulation in neurons, microglia, or other glial populations. Combining these approaches with functional assessments may help identify strategies that limit pathological inflammatory signaling while preserving essential neuroprotective and metabolic functions. Ultimately, the therapeutic potential of CaMKK2 modulation will depend on whether selective interventions produce reproducible effects across relevant CNS disease models and under defined cellular and pathological conditions.

## 9. Conclusions

Across CNS disorders, CaMKK2-associated effects and the level of mechanistic support vary substantially between experimental settings, as summarized in Table 1.

Evidence for a predominantly neuroprotective role of CaMKK2-associated signaling is strongest in adult ischemic stroke, where findings from multiple experimental approaches are largely consistent. In neonatal hypoxic–ischemic injury, the effects of AMPK modulation depend on intervention timing, whereas the temporal and cell type-specific effects of CaMKK2 modulation remain unclear. In glioblastoma, in vivo studies indicate that neuronal CaMKK2 can promote an immunosuppressive microenvironment and contribute to resistance to immune checkpoint blockade. By contrast, tumor-cell-associated findings vary across studies, and whether molecular subtype contributes to these differences remains unresolved. Evidence linking CaMKK2 to psychiatric disorders remains mixed, with genetic associations, functional studies, and behavioral findings providing limited support for disease-specific causal roles. In PD, the evidence is more limited and comes mainly from a specific intervention model and transcriptomic or bioinformatic analyses. More broadly, the functions of CaMKK2 in specific cell types and their variation across disease stages remain incompletely understood. In particular, whether CaMKK2-dependent signaling in astrocytes, microglia, and oligodendrocyte-lineage cells contributes to neuroinflammation and neuron-glia interactions requires further experimental validation.

The context-dependent effects of CaMKK2 highlight several areas for further investigation. First, cell type-specific and inducible genetic models, together with rescue experiments, are needed to clarify the contributions of CaMKK2 in neurons, endothelial cells, and glial populations to disease outcomes. Second, temporally controlled interventions should be used to determine whether the effects of CaMKK2 manipulation differ across stages of injury, particularly in neonatal HI, and to distinguish these effects from those mediated by downstream AMPK signaling. Third, the development of more selective CaMKK2 inhibitors should be accompanied by assessments of unbound brain exposure and direct target engagement in relevant CNS cell populations. Finally, pharmacodynamic biomarkers are needed to distinguish CaMKK2 inhibition from changes in AMPK signaling driven by other upstream pathways. These approaches will help establish whether CaMKK2 can be safely and effectively targeted in specific CNS disorders.

## Figures and Tables

**Table 1:** Context-dependent roles and therapeutic challenges of CaMKK2 signaling in CNS disorders.

Disease Context (References)	Representative Signaling/Evidence	Main Finding/Interpretation	Key Unresolved Issue	Evidence Basis
Adult ischemic stroke [46,47,49,50,54,55,56]	Endothelial CaMKK2-BBB; H_2_S-CaMKK2-AMPK; AMPK-PGC-1α; STC-1/OL-FS13-associated pathways	CaMKK2 loss worsens injury; several protective inflammatory and metabolic pathways lack full CaMKK2-specific validation.	Cell-specific neurovascular mechanisms and downstream pathway dependence.	F; P
Neonatal hypoxic–ischemic injury [60,61,64,65]	CaMKK2 deficiency and post-insult STO-609 treatment; CaMKK2-AMPKα1 activation; timing-dependent STO-609 effects in neuronal OGD models	CaMKK2 loss worsens injury in vivo; neuronal OGD results do not establish opposing CaMKK2-specific timing effects.	Temporal CaMKK2 effects, AMPK isoforms, and proposed AMPK-Nrf2 involvement.	F; P
Depression and anxiety [6,70,71,72]	D-mannose-associated CaMKK2/LKB1-AMPK-ACSS2 signaling; CaMKK2 T85S variant; behavioral phenotypes in CaMKK2-deficient mice	Behavioral and signaling findings suggest involvement in affective regulation, not established human disease causality.	CaMKK2 versus LKB1 contributions; disease-relevant neural circuits.	F; P; A
Bipolar disorder [3,6,17,75,76]	GSK3-mediated CaMKK2 regulation; *CAMKK2* variants; behavioral phenotypes in CaMKK2-deficient mice	Variant-function and mouse studies suggest a role in affective regulation; lithium mediation and disease causality remain unproven.	Bipolar-specific mechanisms and CaMKK2 involvement in lithium responses.	F; P; A
Alzheimer’s disease [79,80,82,83,85]	Aβ-CaMKK2-AMPK signaling involving Tau, MFF, and ULK2; RAGE-CaMKK2-AMPK-associated autophagosome formation; miR-9 regulation; reduced CaMKK2 in postmortem cortex	Experimental evidence supports selected Aβ-related synaptic and mitochondrial effects; reduced postmortem expression is associative.	Autophagic flux, glial inflammation, and human expression findings.	F; P; A
Parkinson’s disease [89,90,91,93,94]	Microglial TRPV1-Ca^2+^-CaMKK2-AMPK/mTOR signaling; transcriptomic *CAMKK2* associations and predicted miRNA regulation	One intervention model links this pathway to α-synuclein clearance; direct CaMKK2 dependence remains uncertain.	Genetic validation of α-synuclein clearance and autophagic flux.	P; A
Glioblastoma [102,103,104,105]	Tumor-cell *CAMKK2* expression and functional perturbation; miR-1271/CaMKK2 and Akt-associated signaling; neuronal CaMKK2-dependent modulation of the tumor microenvironment	Functional host-neuronal and tumor-cell studies indicate distinct roles; clinical expression associations do not prove causality.	Compartment-specific mechanisms and conflicting expression patterns.	F; P; A

Abbreviations: Aβ, amyloid-β; ACSS2, acyl-CoA synthetase short-chain family member 2; Akt, protein kinase B; AMPK, AMP-activated protein kinase; BBB, blood–brain barrier; CaMKK2, calcium/calmodulin-dependent protein kinase kinase 2; GSK3, glycogen synthase kinase 3; H_2_S, hydrogen sulfide; LKB1, liver kinase B1; MFF, mitochondrial fission factor; miR, microRNA; miRNA, microRNA; mTOR, mechanistic target of rapamycin; Nrf2, nuclear factor erythroid 2-related factor 2; OGD, oxygen-glucose deprivation; PGC-1α, peroxisome proliferator-activated receptor gamma coactivator 1α; RAGE, receptor for advanced glycation end products; STC-1, stanniocalcin-1; TRPV1, transient receptor potential vanilloid 1; ULK2, Unc-51-like autophagy activating kinase 2. Note: F, functional evidence involving CaMKK2-directed genetic or biochemical perturbation; P, pathway-level evidence without complete CaMKK2-specific validation; A, associative evidence from genetic, expression, transcriptomic, or bioinformatic analyses. Multiple categories can apply. These codes describe evidence types, not formal grades of study quality or proof of disease causality. Findings based solely on nonselective inhibitors do not establish CaMKK2 dependence.

**Table 2:** Current pharmacological tools for CaMKK2 inhibition and their translational limitations.

Pharmacological Tool	Key Pharmacological Features	Quantitative Pharmacological Parameters	Major Limitation	Key References
STO-609	Widely used in cellular and animal models; reported off-targets include CK2, ERK8, MNK1, and PIM3	Enzymatic IC_50_: CaMKK2, 58 nM; CaMKK1, 408 nM. NanoBRET IC_50_: >10 µM	Incomplete kinase selectivity; CNS exposure and target engagement insufficiently characterized	[21,107,108]
GSK650394	Potent inhibitor used in chemical probe development; broad kinase activity, including AMPK, CDK2, and SGK1 in screening assays	Enzymatic IC_50_: CaMKK2, 3 nM; CaMKK1, 33 nM. NanoBRET IC_50_: <3 nM	Extensive off-target activity; CNS exposure and selective engagement unestablished	[106,108]
Compound 4t	Orally bioavailable and brain-penetrant; reduces ghrelin-induced food intake in rodents	CaMKK2 biochemical IC_50_: approximately 6 nM; CaMKK1 and NanoBRET data: NR	CaMKK1 selectivity incompletely characterized; CNS CaMKK2 engagement and disease-model efficacy unestablished	[109]
Compounds 13g and 45	Medicinal chemistry leads with improved kinome selectivity and cellular NanoBRET activity	13g: CaMKK2/CaMKK1 IC_50_, 65/961 nM; NanoBRET, 700 nM. 45: CaMKK2/CaMKK1 IC_50_, 137/1838 nM; NanoBRET, 540 nM	CNS exposure, target engagement, and in vivo disease efficacy unestablished	[106]
SGC-CAMKK2-1	Cellular chemical probe with NanoBRET engagement; negative control available; CaMKK1 remains a target	CaMKK2 enzymatic IC_50_: 30 nM; NanoBRET IC_50_: 270 nM	In vivo pharmacokinetic limitations; CNS target engagement and disease efficacy unestablished	[110]

Abbreviations: AMPK, AMP-activated protein kinase; CaMKK1, calcium/calmodulin-dependent protein kinase kinase 1; CaMKK2, calcium/calmodulin-dependent protein kinase kinase 2; CDK2, cyclin-dependent kinase 2; CK2, casein kinase 2; CNS, central nervous system; ERK8, extracellular signal-regulated kinase 8; IC50, half-maximal inhibitory concentration; MNK1, MAP kinase-interacting serine/threonine-protein kinase 1; NanoBRET, NanoLuc-based bioluminescence resonance energy transfer; NR, not reported; PIM3, PIM serine/threonine kinase 3; SGK1, serum- and glucocorticoid-regulated kinase 1. Note: IC_50_ values were obtained from the indicated publications using different assay systems and should be interpreted in their respective experimental contexts. Enzymatic IC_50_ values reflect biochemical inhibitory potency, whereas NanoBRET IC_50_ values reflect cellular target engagement in the reported assay systems and do not establish target engagement in CNS tissues. Reported off-target kinases represent selected findings from published biochemical or kinase-profiling assays and do not necessarily indicate target inhibition in CNS tissues. Where CNS exposure or target engagement has not been established, this should not be interpreted as evidence of absent brain penetration or activity.

## Data Availability

No new data were created or analyzed in this study. Data sharing is not applicable to this article.
